# Supplementary figures and images for: Vocal constraints on song amplitude in star finches Bathilda ruficauda
Source: PeerJ. 2025 Jul 10;13:e19705. doi: 10.7717/peerj.19705 (PMC12256045; doi:10.7717/peerj.19705)

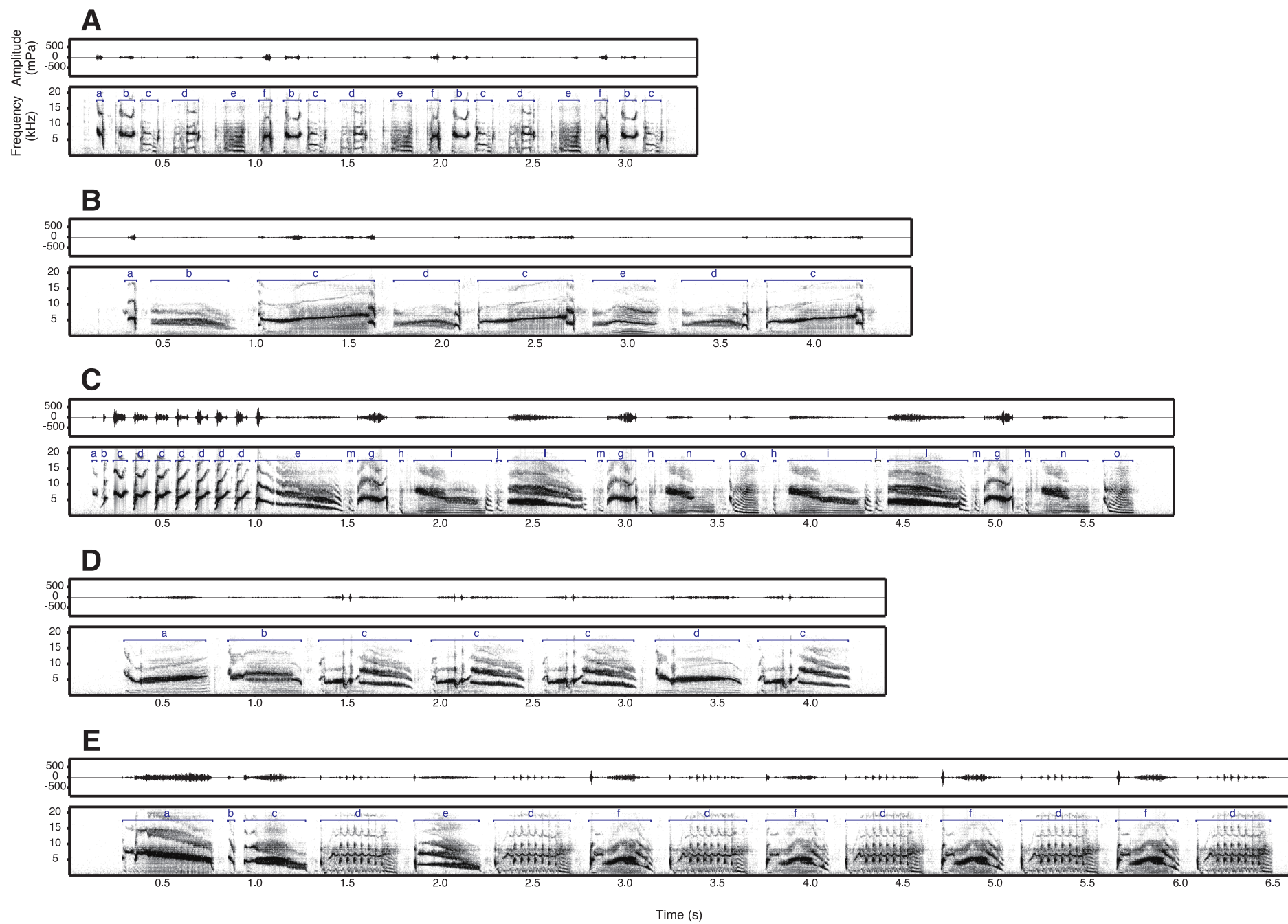

Supplement: Supplemental Information 3 — Each panel shows the song type of one individual male ((A) SF0018, (B) SF0034, (C) SF0042, (D) SF0045, (E)SF0044; bird IDs correspond to Fig. 2). The syllable types are shown in dark blue. [file peerj-13-19705-s003.pdf]

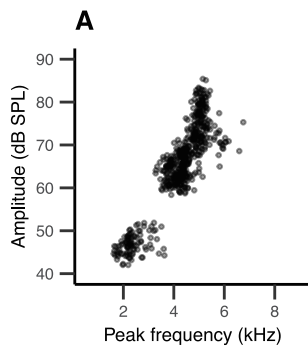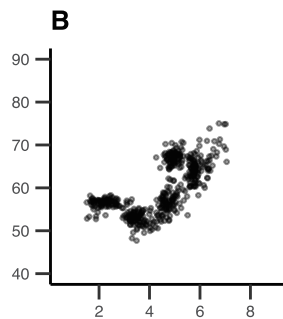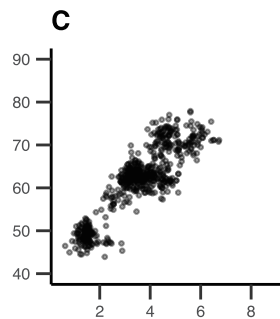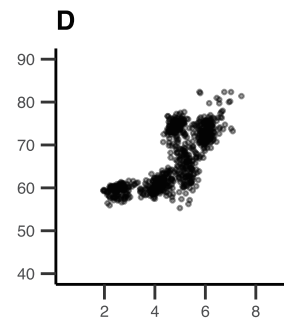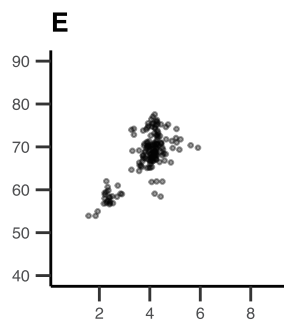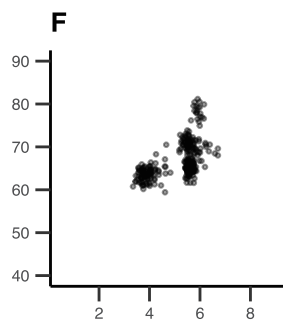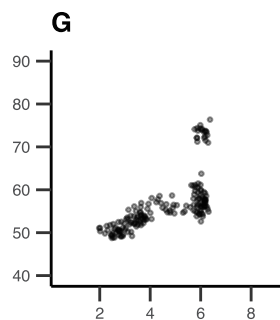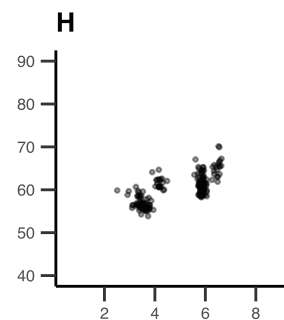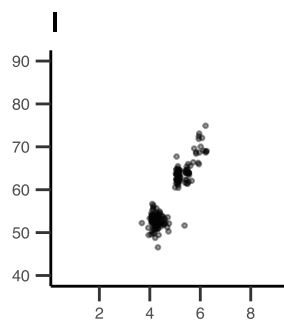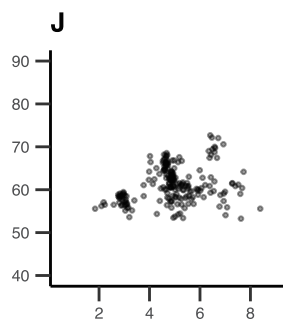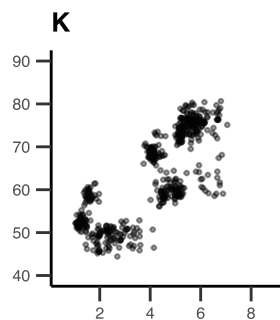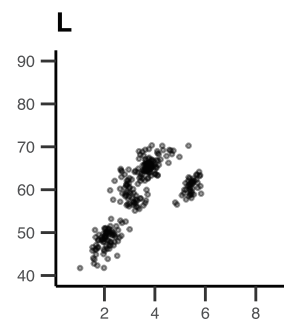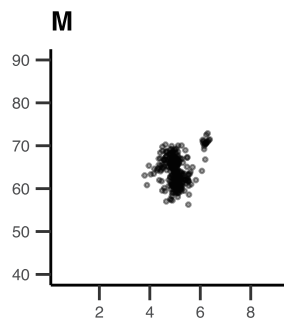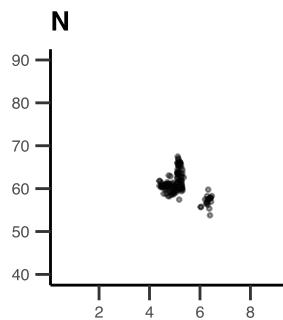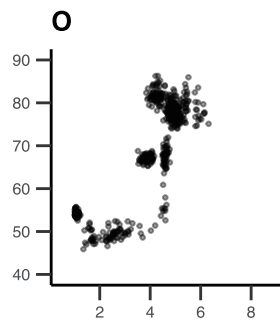

Supplement: Supplemental Information 4 — Each panel shows the syllable amplitudes across peak frequencies for the songs of one male ((A) SF0002, (B) SF0018, (C) SF0020, (D) SF0025, (E) SF0026, (F) SF0029, (G) SF0031, (H) SF0032, (I) SF0034, (J) SF0037, (K) SF0042, (L) SF0043, (M) SF0044, (N) SF0045, and (O) SF0046; bird IDs correspond to Fig. 2). The amplitude values were measured at around 11cm above the birds. Each dot represents a single syllable. [file peerj-13-19705-s004.pdf]
